# Asymptomatic bacteriuria in sickle cell disease: a cross-sectional study

**DOI:** 10.1186/1471-2334-6-46

**Published:** 2006-03-15

**Authors:** Vanessa Cumming, Susanna Ali, Terrence Forrester, Karen Roye-Green, Marvin Reid

**Affiliations:** 1 Sickle Cell Unit, Tropical Medicine Research Institute, University of the West Indies, Mona Campus, Kingston 7, Jamaica; 2 Department of Microbiology, University Hospital of the West Indies, Mona, Kingston 7, Jamaica

## Abstract

**Background:**

It is known that there is significant morbidity associated with urinary tract infection and with renal dysfunction in sickle cell disease (SCD). However, it is not known if there are potential adverse outcomes associated with asymptomatic bacteriuria (ASB) infections in sickle cell disease if left untreated. This study was undertaken to determine the prevalence of ASB, in a cohort of patients with SCD.

**Methods:**

This is a cross-sectional study of patients in the Jamaican Sickle Cell Cohort. Aseptically collected mid-stream urine (MSU) samples were obtained from 266 patients for urinalysis, culture and sensitivity analysis. Proteinuria was measured by urine dipsticks. Individuals with abnormal urine culture results had repeat urine culture. Serum creatinine was measured and steady state haematology and uric acid concentrations were obtained from clinical records. This was completed at a primary care health clinic dedicated to sickle cell diseases in Kingston, Jamaica. There were 133 males and 133 females in the sample studied. The mean age (mean ± sd) of participants was 26.6 ± 2.5 years. The main outcome measures were the culture of ≥ 10^5 ^colony forming units of a urinary tract pathogen per milliliter of urine from a MSU specimen on a single occasion (probable ASB) or on consecutive occasions (confirmed ASB).

**Results:**

Of the 266 urines collected, 234 were sterile and 29 had significant bacteriuria yielding a prevalence of probable ASB of 10.9% (29/266). Fourteen patients had confirmed ASB (prevalence 5.3%) of which 13 had pyuria. Controlling for genotype, females were 14.7 times more likely to have confirmed ASB compared to males (95%CI 1.8 to 121.0). The number of recorded visits for symptomatic UTI was increased by a factor of 2.5 (95% CI 1.4 to 4.5, p < 0.005) but serum creatinine, uric acid and haematology values were not different in patients with confirmed ASB compared with those with sterile urine. There was no association with history of gram negative sepsis.

**Conclusion:**

ASB is a significant problem in individuals with SCD and may be the source of pathogens in UTI. However, further research is needed to determine the clinical significance of ASB in SCD.

## Background

The definition of asymptomatic bacteriuria (ASB) is controversial as some have defined it as the quantitative growth of bacteria, greater than or equal to 10^5 ^colony forming units per milliliter urine of the same organism, on aseptically collected midstream urine specimens, in the absence of symptoms of urinary tract infection on two or more consecutive occasions [1,2] while for others a single occasion is sufficient [3-5]. The prognostic significance of ASB resides in the observation that persons with ASB in certain medical conditions, such as diabetes mellitus and pregnancy, are at increased risk of pyelonephritis and renal impairment [5-7].

In sickle cell disease renal disease and dysfunction are common and increase morbidity and mortality [8,9]. Additionally, symptomatic urinary tract infection (UTI) is associated with painful crisis, bacteraemia, pneumonia, and osteomyelitis in SCD [10-13]. Whether ASB is a prelude to more serious renal disease and dysfunction in SCD is unclear. This present study was undertaken to estimate the prevalence and possible risk factors of ASB in the Jamaican sickle cell cohort.

## Methods

### Patients

The sample comprised a group of patients in the Jamaican Sickle Cell Cohort attending the 2004 Annual Sickle Cell Cohort Review conducted at the Sickle Cell Unit Clinic at the University of the West Indies, Kingston, Jamaica. The Jamaican Sickle Cell Cohort includes patients with sickle cell diseases detected using standard criteria during screening of 100,000 consecutive non-operative deliveries at a large maternity hospital (the Victoria Jubilee Hospital) between 1973 and 1981. This screening yielded 580 patients with different sickle genotypes, of which 315 had HbSS disease, 201 Sickle cell-haemoglobin C, 33 Sickle cell-β^+ ^thalassaemia, 14 Sickle cell-β^0 ^thalassaemia and 17 sickle variants. They have been followed clinically since birth [14]. At the 2004 cohort review 267 of 302 patients eligible to attend the annual cohort review were seen. The reasons for being absent were: Twenty-two of the patients did not attend the review that year, 11 were lost to follow up, or incarcerated, and 2 were ill at the time of review. One patient refused to leave a sterile urine sample during the cohort review period, thus the final participating patient sample for this study was 266 patients. Ethical approval was granted for this study by the University Hospital of the West Indies, University of the West Indies Faculty of Medical Sciences Ethics Committee.

### Measurements

Aseptically collected midstream urines (MSUs) were obtained from the symptom free attendees of 2004 cohort review and sent on the same day for microbiological culture. Urinalysis was performed on aliquots of the MSU specimen using the QuickVue UrinChek™10^+^SG test strips. Bacterial culture was performed by streaking 0.002 mL of midstream collected urine with a calibrated loop on MacConkey and 5% sheep blood agar plates. These agar plates were incubated at 35°C for 24 hours under aerobic conditions. Isolates were considered significant if there were ≥ 10^5 ^colony forming unit/mL (CFU/mL) with 2 or less isolates, doubtful significance if 10^4 ^– 10^5 ^CFU/mL, insignificant if < 10^4 ^CFU/mL. Mixed growths, in any count, of more than two organisms were considered to be contaminated. Significant isolates were selected for identification and antimicrobial susceptibility testing using the vitek (biome'rieux' version R06.01, Missouri USA). Proteinuria was defined as a protein reading of trace or greater on dipstick urinalysis.

Patients complaining of any symptoms, whether related or unrelated to the urinary tract, were not included in the study on that day. Rather, these patients were treated for their complaint and studied on another day when they were without symptoms. Subjects whose urine microbiological report was abnormal were asked to return to the unit for a repeat midstream urine collection and culture.

Blood samples were also taken for the measurement of serum creatinine. Serum creatinine were measured in the Tropical Medicine Research Institute laboratory with an Abbott ALCYON 300I, using the alkaline picrate method.

The clinical records of all cohort patients in the sickle cell unit database were examined and the following information extracted: age, gender, steady state haematology, steady state uric acid levels, history of urinary tract infection, and history of gram negative sepsis.

### Definition of asymptomatic bacteriuria

Probable ASB was defined as the presence of at least 10^5 ^colony forming units of a urinary tract pathogen per milliliter of urine in a culture of a midstream urine specimen obtained from a patient during the cohort review visit. A confirmed episode of asymptomatic bacteriuria was defined as two or more consecutive cultures with evidence of asymptomatic bacteriuria due to the same urinary tract pathogen with the same sensitivity pattern. A sample was deemed grossly contaminated if it grew more than two urinary tract pathogens even if in significant quantities.

### Statistical methods

Values are expressed as counts or means ± sd as appropriate. The prevalence of probable ASB and confirmed ASB were determined as the ratio of the number of urines classified as probable ASB and confirmed ASB, to the total number of collected MSU samples. The 95 percent confidence interval (95%CI) for this proportion was computed according to the Wilson score method without continuity correction [15]. For continuous outcome variables differences in means between ASB status (confirmed ASB group vs. sterile urine group) and genotype were determined by two factor analysis of variance. Associations between categorical exposures and ASB status were adjusted for genotype by Mantel-Haenszel methods. Logistic regression was used to examine the relationship between potential predictors (age, history of urinary tract infection, genotype, steady state haematology, uric acid and serum creatinine) and probability of having ASB. Likelihood ratio tests were used to determine which predictors would be included in the final model. We assessed differences in the frequency of past attendance for symptomatic urinary tract infection between ASB status and genotype using a negative binomial regression model. Clinic visits less than 14 days apart were considered the same event. Visits were assumed to be independent between individuals but repeat visits by individuals were not assumed to be independent. Data were analyzed using the Stata statistical software version 8 (Statacorp, TX, USA). Tests were considered significant if p < 0.05.

## Results

**Figure 1:**
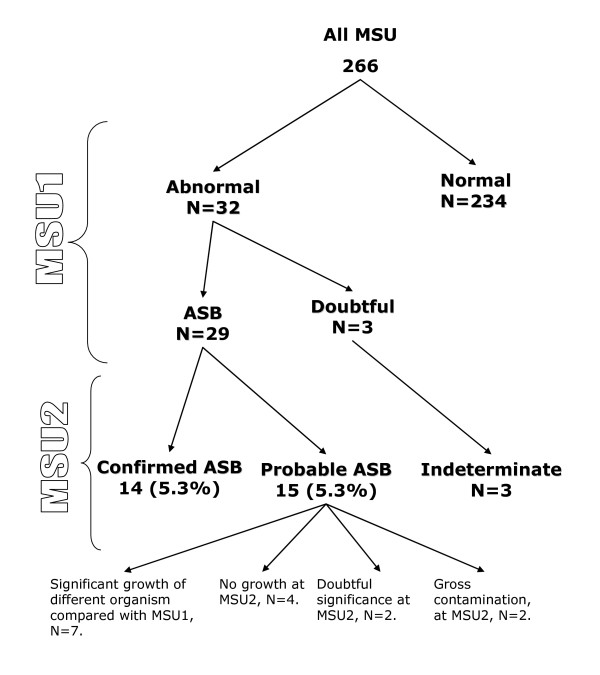
Number of urine samples by microbiological classification.

A total of 267 patients attended the 2004 cohort review. One person refused to participate in the study. Thus 266 patients had screening clean-catch MSU (Figure 1), of which 50% were male. One woman was pregnant at the cohort review visit; and another became pregnant during follow-up of the first abnormal MSU.

As expected there were significant differences in the haematological values by genotype with the mean steady state haemoglobin levels of HbSS group being significantly lower than sickle cell-haemoglobin C group, sickle cell-β^+ ^thalassaemia, and sickle variants but having higher steady state nucleated cell counts (Table 1). The steady state serum creatinine values were significantly lower in the HbSS group compared with other genotypes except the sickle variant group. In addition the mean uric acid values were lower in the HbSS group compared with sickle cell-β^+ ^thalassaemia group (Table 1).

**Table 1:** Clinical Characteristics of enrolled subjects by genotype (N = 266).

Clinical characteristics	HbSS N = 148	HbSC N = 85	HbSβ^0 ^N = 6	HbSβ^+ ^N = 22	Sickle variants* N = 5
Age yr	26.4 ± 2.6	27 ± 2.3	25.9 ± 2.3	26.5 ± 2.5	25.1 ± 2.5
†Gender (M:F)	71:77	38:47	2:4	13:9	4:1
#Creatinine μmol/L	45.7 ± 9.4^a^	63.3 ± 11.7^b^	70.7 ± 23.1^b^	61.9 ± 12.4^b^	62 ± 20.4^a^
#Uric Acid mmol/L	0.3 ± 0.1^a^	0.3 ± 0.1^a^	0.4 ± 0.1^a^	0.4 ± 0.3^b^	0.3 ± 0.1^a^
#Haemoglobin g/L	80.2 ± 9.9^a^	108.2 ± 8^b^	91.6 ± 11.8^b^	109.9 ± 12.4^b^	124 ± 22.1^b^
#Nucleated Blood cells × 10^9^/L^c^	15.9 ± 3.2^a^	10.2 ± 2.9^b^	13.3 ± 2.2^b^	9.2 ± 2.7^b^	9.9 ± 2.5^b^
†Proteinuria ≥ trace : No proteinuria (n = 182)	17	7	0	1	0
†Positive History of gram negative sepsis	2	2	0	0	0
†Positive recorded history of ever having UTI	50	25	2	1	1

Values are means ± sd ; † values are counts. Abbreviations – HbSS = Homozygous sickle cell disease, HbSC= Sickle cell-haemoglobin C, HbSβ^0 ^= Sickle cell-β^0 ^thalassemia, HbSβ^+ ^= Sickle cell-β^+ ^thalassemia; UTI= Urinary Tract Infections.

* Sickle variants include sickle cell-hereditary persistence of foetal haemoglobin, sickle cell -haemoglobin O Arab, sickle cell -haemoglobin Lepore-Boston.

# Significant difference in mean values by genotype. Dissimilar superscript to HbSS values are significantly different (p < 0.03)

There were 32 patients with abnormal first MSUs: 29 were positive MSU samples growing identifiable pathogens (Figure 1), 2 were grossly contaminated and one grew bacteria of doubtful significance (growth of a urinary tract pathogen between 10^4 ^and 10^5 ^per mL). Of the 29 positive cultures 15 re-grew the same organisms in the next culture but one of these grew the same organism (*E. coli*) but with a different sensitivity pattern and was therefore classified as a probable ASB. Thus 14 samples satisfied the criteria for a confirmed episode of asymptomatic bacteriuria (Figure 1) yielding a prevalence of 5.3% (95% CI 0.8% to 29.4%). Fifteen subjects were classified as probable ASB. The probable ASB group consisted of seven samples which grew organisms that were different from the first MSU sample, 2 grew bacteria in insignificant quantities, four were no growth and two were grossly contaminated specimens (Figure 1). None of the patients were on antibiotic therapy during this study and all of the patients with identified ASB had at least one follow-up visit.

Sixteen of the 29 positive first MSU samples (55%) grew *E. coli *in significant quantity. This was the commonest organism cultured in this study. Other urinary tract pathogens identified in this study included: group B *Streptococcus*, *Enterobacter aerogenes, coagulase negative Staphylococcus, Enterobacter cloacae, Proteus mirabilis, Microaerophilic streptococcus *and *Bacteroides *(mixed), group B *Streptococcus *and *Streptococcus viridans *(mixed), *Enterobacter *species, group D *Streptococcus*, *Klebsiella pneumoniae*, and *Enterobacter koseri*. All of these organisms were grown in pure cultures with only the two exceptions listed above. Fifteen of the patients with probable ASB (52%) had evidence of a host response, as evidenced by pyuria. Of those with confirmed ASB, 13 of 14 patients (93%) had a host response as evidenced by pyuria in the second midstream urinalysis.

There were no significant differences for mean haemoglobin and nucleated blood cell counts between the confirmed ASB group and sterile urine group (86.4 ± 15.2 g/L vs. 92.6 ± 17.4 g/L and 14.0 ± 4.5 × 10^9^/L vs. 13.4 ± 4.1 × 10^9^/L) respectively. Similarly there were no differences in mean serum creatinine and uric acid values between the confirmed ASB group and sterile urine group (Table 2). Twelve and a half percent (1/8) of patients with ASB had proteinuria of trace or greater on dipstick urinalysis. However there was no association between having proteinuria measured by dipstick and ASB (Table 2).

**Table 2:** Clinical characteristics of enrolled subjects with confirmed ASB and sterile urine (N = 248)*

Clinical characteristics	Confirmed ASB N = 14	Sterile urine N = 234
Age yr	27.9 ± 2.3	26.4 ± 2.5
†Gender (M:F)	1:13	124:110
#Genotype frequency (HbSS:HbSC:HbSβ^0^:HbSβ^+^: sickle variants)	9:2:1:2:0	130:75:5:19:5
Creatinine μmol/L	49.1 ± 12.4	53.7 ± 14.3
Uric Acid mmol/L	0.36 ± 0.29	0.33 ± 0.08
†Proteinuria ≥ trace : No proteinuria (n = 168)	1:7	22 :138
†Positive History of gram negative sepsis	1	1
†Positive recorded history of ever having UTI	7	25
†Cumulative number of recorded clinic visits for symptomatic UTI*	18	49

*Values of subjects with probable ASB excluded from table. † Values are counts. Abbreviations : ASB = asymptomatic bacteriuria; HbSC= Sickle cell-haemoglobin C, HbSβ^0 ^= Sickle cell-β^0 ^thalassemia, HbSβ^+ ^= Sickle cell-β^+ ^thalassemia; UTI = Urinary Tract Infections

#Sickle variants include sickle cell-hereditary persistence of foetal haemoglobin, sickle cell -haemoglobin O Arab, sickle cell -haemoglobin Lepore-Boston;

The proportion of subjects with HbSS in the confirmed ASB group (64%) was not statistically different from the proportion of HbSS in the sterile group (55%). However controlling for genotype, females were 14.7 times more likely to have confirmed ASB compared to males (95%CI 1.8 to 121.0) but having a recorded history of ever having a symptomatic UTI was not associated with current ASB status (Odds ratio 2.5, 95%CI 0.79 to 7.76). The numbers of recorded episodes of gram negative sepsis were few. Notwithstanding, there were 148 clinic visits for symptomatic urinary tract infections (Table 2). Compared to the sterile urine group the expected number of visits for symptomatic UTI in the confirmed ASB group is increased by a factor of 2.5 (95% CI 1.4 to 4.5, p < 0.005).

The relationship between the various predictors and probability of having ASB were explored with a series of logistic regression models. Likelihood ratio tests were used to determine which variables should be incorporated into a final regression model with ASB as the response variable. The results indicated that the absence of the variables HbSS genotype (coded as 1 for homozygous β^s^, 0 otherwise), steady state haematology, and serum creatinine from the full model which included these variables along with age, gender (coded as 1 for female, 0 otherwise) and steady state uric acid concentrations as predictors did not significantly reduce the log likelihood. Thus the model which best predicted ASB from the data included the predictors age OR 1.37 (95 % CI: 0.99 1.64), female gender OR 15.3 (95% CI: 1.94, 120.1), and uric acid concentration OR 21.5 (95% CI: 0.64, 722.2) (Table 3).

**Table 3:** Model predicting asymptomatic bacteriuria in the Jamaica Sickle Cell Cohort.

Variables	Odds ratio	P value	Lower 95% CI	Upper 95% CI
Age years	1.37	0.057	0.99	1.64
Female (coded as 1)	15.3	0.010	1.94	120.12

Uric acid mmol/l	21.5	0.087	0.64	722.21

## Discussion

The prevalence of probable ASB was 10.9% and the prevalence of confirmed ASB was 5.3% with 95% CI 0.8% to 29.4% in this sample of Sickle Cell patients. These prevalence rates are comparable to other prevalence estimates of ASB in non-sickle cell healthy populations [5,16]. The major determinants of ASB status were gender, age and steady state serum uric acid concentrations. Additionally there were increased numbers of recorded clinical visits for symptomatic UTI in the confirmed ASB group. The effect of genotype status on these relationships was not statistically significant.

The strength of this study is related to the cohort design which resulted in the selection of a representative population based sample. This reduces the ascertainment bias associated with symptomatic recruitment from a clinic based population. In addition, the participation rates at this cohort review were >99%, thus strengthening our confidence in extrapolating our findings to the wider sickle cell population. However the small number of patients identified with confirmed ASB represents a limitation of the present study.  This was partly due to our conservative definition of confirmed ASB. The consequence of having a small numerator is that the precision of our prevalence estimate is affected and this is reflected in a wide 95% confidence interval.

The sexual dimorphism in the prevalence of ASB has been reported before in healthy adults [6]. It is thought that this may be related a relative deficiency of secretory IgA antibody response from the mucosal surface in the urogenital tract of females compared with males [6].

In non pregnant females the presence of confirmed ASB has a high sensitivity and specificity of predicting a future symptomatic episode of UTI [5,6]. However in the absence of anatomical or functional abnormalities of the urinary tract, ASB per se is not associated with renal scarring, renal dysfunction or hypertension [17]. In adults with SCD, anatomical abnormalities such as renal cortical scarring, enlarged glomeruli, vascular disorganization of the medulla and renal functional abnormality such as increased effective renal blood flow and hyperfiltration are common [8]. These pathophysiological changes are thought to contribute to sickle cell nephropathy and the high prevalence of renal failure observed in subjects with sickle cell disease [8]. In this context, one might expect persons with ASB to have more UTI and worse renal function. Thus the finding of an increased number of expected visits for symptomatic UTI in subjects with ASB compared to those without as well a trend for a significant association between ASB status and a history of UTI is not surprising. On the other hand, the absence of significant differences in measures of renal function between those with ASB and those without ASB was unexpected. However, serum creatinine and uric acid are not sensitive measures of renal function as glomerular filtration rate has to fall by up to 50% before there are changes in serum creatinine [18,19]. Additionally variation in creatinine production due to differences in muscle mass may alter serum creatinine levels independent of renal functional status [20,21]. Notwithstanding, further prospective studies are needed to define the renal risk posed by ASB in sickle cell disease and to inform therapeutic strategies.

In sickle cell disease, the increased purine turnover associated with accelerated erythropoesis results in the greater production of uric acid [22]. This is counterbalanced by increased renal clearance of urate. It is possible that the increased renal clearance may play a predisposing role for ASB by promoting the formation of microcrystals which damage the uroepithelium thereby facilitating colonization.

The infecting organisms identified in this study are in keeping with commonly isolated bacteria in other studies [1-3,5,10,11]. The presence of bacteria in the urine in the absence of a major inflammatory response and hence symptoms, suggest that there are alterations in the host-pathogen interaction. For example it has been reported that uropathogenic strains of microbes have evolved mechanisms to promote survival in the urinary space. Features of these adaptive mechanisms include presence of adhesion which promote attachment to the urogenital tract, and the production of factors such as α-haemolysin, and cytotoxic necrotizing factor 1 (CNF1) [6]. On the other hand the host factors in SCD that increases susceptibility to bacterial colonization are unclear but in Diabetes Mellitus altered leukocyte function contribute to the susceptibility to ASB in this disease [23].

## Conclusion

In summary ASB is a significant problem in individuals with SCD and is associated with UTI. However, further research is needed to determine the clinical and renal significance of ASB in SCD.

## Abbreviations

SCD: sickle cell disease, ASB: asymptomatic bacteriuria, MSU: midstream urine, UTI: urinary tract infection, CFU: colony forming unit.

## Competing interests

The author(s) declare that they have no competing interests.

## Authors' contributions

SA and MR coordinated and supervised the cohort review at the Sickle Cell Clinic; VC collected data, managed the patients with ASB, and wrote the paper in collaboration with TF, MR and KRG. All authors read and approved the final manuscript.

## Pre-publication history

The pre-publication history for this paper can be accessed here:


